# Retraction of: P-1833. Acute Viral Hepatitis in Hospitalized Children: Clinico-Biochemical, Ultrasonographic and Etiological Findings from North India

**DOI:** 10.1093/ofid/ofag306

**Published:** 2026-06-11

This is a retraction of Monirujjaman Biswas, P-1833. Acute Viral Hepatitis in Hospitalized Children: Clinico-Biochemical, Ultrasonographic and Etiological Findings from North India, *Open Forum Infectious Diseases*, Volume 13, Issue Supplement_1, January 2026, ofaf695.2002, https://doi.org/10.1093/ofid/ofaf695.2002.

The journal is retracting this IDWeek abstract because there is significant overlap between it and a previously published abstract without attribution:

Clinico-biochemical, ultrasonographic profile and etiology of acute viral hepatitis in hospitalized children: A cross-sectional study (https://ijlbpr.com/htmlissue.php?id=3052). Additionally, the authorship of the publication cannot be verified.

